# Impacts of leguminous shrub encroachment on neighboring grasses include transfer of fixed nitrogen

**DOI:** 10.1007/s00442-015-3538-5

**Published:** 2016-01-08

**Authors:** Hai-Yang Zhang, Qiang Yu, Xiao-Tao Lü, Susan E. Trumbore, Jun-Jie Yang, Xing-Guo Han

**Affiliations:** State Key Laboratory of Forest and Soil Ecology, Institute of Applied Ecology, Chinese Academy of Sciences, Shenyang, 110164 China; Max Planck Institute for Biogeochemistry, 07745 Jena, Germany; Department of Biology, Graduate Degree Program in Ecology, Colorado State University, Fort Collins, CO 80523 USA

**Keywords:** Species-specific, Nitrogen transfer, Shrub encroachment, Plant facilitation, Plant competition

## Abstract

Shrub encroachment induced by global change and human disturbance strongly affects ecosystem structure and function. In this study, we explore the degree to which invading leguminous shrubs affected neighboring grasses, including via the transfer of fixed nitrogen (N). We measured N concentrations and natural abundance ^15^N of shoot tissues from three dominant grasses from different plant functional groups across seven distances along a local transect (up to 500 cm) to the leguminous shrub, *Caragana microphylla*. *C. microphylla* did transfer fixed N to neighboring grasses, but the amount and distance of N transferred were strongly species-specific. Shoot N concentrations decreased significantly with distance from *C. microphylla*, for a rhizomatous grass, *Leymus chinensis*, and a bunchgrass, *Achnatherum sibiricum*. However, N concentrations of another bunchgrass, *Stipa grandis*, were higher only directly underneath the shrub canopy. Shoot δ^15^N values of *L. chinensis* were enriched up to 500 cm from the shrub, but for *S. grandis* were enriched only below the shrub canopy. In contrast, δ^15^N of *A. sibiricum* did not change along the 500-cm transect. Our results indicated the rhizomatous grass transferred fixed N over long distances while bunchgrasses did not. The presence of *C. microphylla* increased the shoot biomass of *L. chinensis* but decreased that of *S. grandis* and *A. sibiricum*. These findings highlight the potential role of nutrient-acquisition strategies of neighboring grasses in moderating the interspecific variation of fixed N transfer from the leguminous shrub. Overall, leguminous shrubs have either positive or negative effects on the neighboring grasses and dramatically affect plant community composition and structure.

**Electronic supplementary material:**

The online version of this article (doi:10.1007/s00442-015-3538-5) contains supplementary material, which is available to authorized users.

## Introduction

Global change and human disturbance and their complex interactions have induced indigenous shrub encroachment in arid and semi-arid grasslands (Schlesinger et al. 1990; Archer et al. 1995; D’Odorico et al. 2012). Previous studies showed that positive and negative interactions exist between encroaching shrubs and neighborhood grasses under field conditions (Scholes and Archer 1997). Many of the woody shrubs that encroach in arid or semiarid grasslands are leguminous species which form symbiosis with dinitrogen-fixing bacteria (Knapp et al. 2008; Eldridge et al. 2011), thereby potentially facilitating neighboring plants by providing additional N inputs to the ecosystem (Carlsson and Huss-Danell 2003). Fixed N can be transferred from the legume to a non-legume species (Sierra and Nygren 2006), and this additional N source may disrupt the natural competitive balance among neighboring native grasses, endangering inferior competitors in nutrient-poor ecosystems (Wedin and Tilman 1993; Hellmann et al. 2011; Soudzilovskaia et al. 2012). However, whether fixed N from leguminous shrub can be transferred to neighboring grasses under field conditions and how these processes operate among different grasses remains unclear.

Nitrogen limits plant growth in most terrestrial ecosystem (Vitousek and Howarth 1991) and plays a critical role in shaping plant community structure (Tilman 1988; Yu et al. 2010) and plant–soil feedbacks (Vivanco and Austin 2011). However, the role of leguminous shrubs goes beyond N subsidies to soil, because they may also suppress growth of co-occurring grasses by increasing shading, reducing water availability, and/or inhibiting germination (Walker and Vitousek 1991; Soudzilovskaia et al. 2012). Facilitation and competition between plants play an essential role in shaping community structure (Callaway et al. 2002). However, the net effects of leguminous shrub encroachment on the biomass of different grasses and plant community composition under field conditions remain unknown.

Laboratory studies demonstrated that N transfer between plants strongly depended on their N acquisition strategies, suggesting N transfer between legumes and non-legumes should be species-specific: plants with cluster-roots or infected with ectomycorrhizal fungi transfer more N than those associated with arbuscular mycorrhizal fungi (Teste et al. 2015). Besides affecting the amount of transferred-N, the transfer distance of fixed N from legume to non-legume might also differ by species. For example, rhizomatous plants that share resources among connected ramets (Stuefer et al. 1994; Alpert 1996) could extend the distance of fixed N transfer. Overall, coexisting neighboring species with contrasting root traits have different nutrition uptake strategies (Liu et al. 2015) and should demonstrate strong differences in the amount and the distance of fixed N transfer following shrub encroachment. More empirical evidence is needed to confirm the role of species identity in mediating N-transfer between legumes and non-legumes.

Several studies have estimated the fixed N transfer between legume and non-legume species growing together (Ledgard 1991; Chalk et al. 2014). Sierra and Nygren (2006) showed that about one-third of the total N in non-legume grasses was transferred from neighboring legumes by tracking movement of an enriched ^15^N label. But even with the help of N isotopic tracer (enriched ^15^N labeling either the soil or plants) (Chalk 1996; Chalk et al. 2014), reliably quantifying the fixed N transfer remains difficult and uncertain (Høgh-Jensen 2006).

The ^15^N natural abundance method has also been used to investigate fixed N transfer (Peoples et al. 2015). Legume species typically contain higher tissue N, have δ^15^N signatures close to the atmospheric N_2_ value (0 ‰), and are less enriched than plants that get their N from soils. Similarly, plants that acquire N transferred from a neighboring legume will have higher N concentrations and lower δ^15^N values compared to neighbors that rely only on the uptake of soil N (Rascher et al. 2012). Simple comparative measures of non-legume ^15^N with or without legume could only suggest the uptake of transferred N but not provide a quantitative estimate of N transfer between plant species if the dominant source and the isotopic identity of the transferred N cannot be validated (Peoples et al. 2015).

In this study, we measured N concentrations and δ^15^N of plant shoot tissues to investigate how three dominant grass species that differ in their N uptake strategies were affected by the legume shrub, *Caragana microphylla.* The original grassland was encroached by the native N_2_-fixing shrub *C. microphylla*, as a consequence of reduced fire and overgrazing (Peng et al. 2013), and this landscape has no other large-stature shrub species. The three dominant grass species included *Leymus chinensis* (Trin.) Tzvel., a perennial rhizomatous species (individuals belowground connected through rhizomes, might share resources among individuals and lengthen the N transfer distance); *Stipa grandis* P. Smirn., a perennial bunchgrass (no direct connection among bunches, might shorten the N transfer distance); and *Achnatherum sibiricum* (Linn.) Keng., another perennial bunchgrass that can be 100 % infected with leaf endophytic fungi (EF) in the field (Wei et al. 2007). Recent studies showed that endophytic fungi assisted their host to take up organic N (Newsham 2011), but their role in determining N transfer between plants remains unclear. We sampled plant communities developed either under the shrub canopy or in nearby open areas that were not influenced by shrubs to examine the effects of the encroaching legume on community structure and composition. We hypothesized that (1) fixed N can be transferred from a leguminous shrub to neighboring grasses, but the amounts and distance of N transfer would be species-specific; specifically, rhizomes would facilitate the transfer of legume fixed N, and thus the rhizomatous species would have higher N concentration and longer transfer distance than these of bunchgrasses; and (2) leguminous shrub encroachment would affect neighboring grasses differently and thus change plant community structure and composition.

## Materials and methods

### Study site

This study was conducted in July 2012 at a permanent field site (43°32′N, 116°40′E, 1200 m a.s.l) near the Inner Mongolia Grassland Ecosystem Research Station (IMGERS). The site has been fenced since 1979 to exclude large animals. Mean annual temperature (averaged over 1982–2005) was 0.3 °C and mean annual precipitation was 346 mm, 60–80 % of which fell during the growing season (May–August). The soil is classified as Calcic-orthic Aridisol according to US Soil Taxonomy and contains 80.2 % sand, 17.6 % silt and 2.2 % clay (top 20 cm) with a soil pH 7.5 (Bai et al. 2010).

### Plant and soil sampling transects

The encroaching *C. microphylla* at this site occurs in multi-individual clusters. During 10–12 July, 2012, we chose six *C. microphylla* clusters with a similar combined canopy size of approximately 66 ± 8 cm (mean ± SD) in height and 243 ± 41 cm in canopy diameter within 4 ha area. Shrub clusters were selected only when there were no other conspecific shrubs within a 10-m radius. Four transects radiating outwards from the canopy center of each target shrub in each cardinal direction were used for sampling of vegetation (Fig. S1). Seven 20 × 20 cm^2^ sampling squares centered at distances of 0, 20, 50, 100, 150, 300 and 500 cm (starting from the edge of the shrub clusters) were placed along each transect. In each quadrat, we clipped the plants at the soil surface and sorted them to species. For each shrub, we grouped the sorted species according to each distance along the four different transects as one replicate, and used the three target grasses for subsequent chemical analyses. Meanwhile, we collected “reference” plant samples: 10 individuals of each grass species were composited for one replicate, with 6 replicates in total, from areas at least 15 m from the closest *C. microphylla* growth, and no other legumes growing within 1 m. To our knowledge, the only other legume existing in our study area is *Melilotoides ruthenica*, which has low density (<5 % of area, as opposed to 40 % for *C. microphylla*), and therefore could be avoided during sampling.

Soil samples were taken from the surface to a depth of 20 cm for each quadrat using a 5-cm-diameter soil auger and samples from the same distance from each of the four transects were homogenized in situ into one composite sample. We also collected “reference” soil samples (6 replicates) from the same areas where we collected our reference plants. Soils were all sampled outside of the shrub canopy and without regard to the species of grass roots that might be contained in them. We also collected non-rhizosphere soil (NRS) and rhizosphere soil (RS) from beneath the canopy of *C. microphylla*. A cube of soil (8000 cm^3^) was excavated from the base of an individual plant within a given cluster, with the plant stem at its center. We carefully shook the plants by hand for 5 min to remove non-adhering soil (e.g., NRS). We considered soil still adhering to the roots after shaking as rhizosphere soil (Wieland et al. 2001); this soil was collected by hand-picking. Soil samples were air-dried, sieved (<2-mm), roots and large organic debris removed by hand, and ~200 g was selected for further processing.

### Plant community sampling

To investigate the response of plant community structure to the shrub invasion, we clipped all the plant species at the soil surface using a 0.5 × 1 m^2^ quadrat frame at two positions: next to the center of the shrub cluster (referred to as “Within”), and an open area approximately 4 m from the edge of the shrub canopy (referred to as “Open”). For the target three grasses, we also recorded the average height and individual numbers in the two comparable plots. The 0.5-m side of the frame was parallel with the transect direction. All plants were sorted by species, oven-dried at 70 °C for 48 h, and then weighed.

### Chemical analysis

Aboveground tissue for each of the three grasses and sieved soil were ground (Retsch^®^ MM200; Hann, Germany). Plant and soil N concentrations and isotopic ratios (i.e. plant δ^15^N) were determined with a combustion analyzer coupled to an isotope-ratio mass spectrometer (IRMS; Deltaplus XP and Delta C prototype Finnigan MAT, respectively; Finnigan MAT, Bremen, Germany; 0.1 ‰ precision). Plant δ^15^N values were calculated as follows: δ^15^N (‰) = (R_sample_/R_standard_–1) × 1000 ‰, where R equals the molar abundance of the heavy isotope divided by the light isotope (^15^N/^14^N). R_sample_ is the sample isotope ratio (^15^N/^14^N) and R_standard_ is the ^15^N/^14^N for atmospheric N_2_.

### Calculation of the proportion of N in neighboring grasses derived from rhizo-deposition transfer

Soil N concentration in the present study was constant not only across the whole transect (0–500 cm distances) but also for the non-rhizosphere and references soils. Only the N concentration for the rhizosphere soil (N_RS_) was greater than non-rhizosphere soil (N_NRS_). We assume that increased N for N_RS_ mainly caused by root deposition (root exudes and root litter input, N_depo_). From this, we could calculate the N proportion of root rhizo-deposition N in rhizosphere soil N (P_depo_):

1 $${\text{P}}_{\text{depo}} = \frac{{({\text{N}}_{\text{RS}} - {\text{N}}_{\text{NRS}} )}}{{{\text{N}}_{\text{RS}} }}$$

The second method for calculating the proportion of rhizo-deposition N in rhizosphere soil N was using a mixing model equation (Chalk et al. 2014):

2 $${\text{P}}_{\text{depo}} = \frac{{\left( {\delta^{15} {\text{N}}_{\text{RS}} - \delta^{15} {\text{N}}_{\text{NRS}} } \right)}}{{\left( {\delta^{15} {\text{N}}_{\text{depo}} - \delta^{15} {\text{N}}_{\text{NRS}} } \right)}}$$

where δ^15^N_depo_ is the natural ^15^N abundance of rhizo-deposition for the concerned leguminous shrub, δ^15^N_NRS_ is the ^15^N value for the non rhizosphere soil of the legume, and δ^15^N_RS_ is the ^15^N value for the rhizosphere soil of the legume. Combining formulas (1) and (2) we had:

3 $$\delta^{15} {\text{N}}_{\text{depo}} = \frac{{\left( {\delta^{15} {\text{N}}_{\text{RS}} - \delta^{15} {\text{N}}_{\text{NRS}} } \right)* {\text{N}}_{\text{RS}} }}{{({\text{N}}_{\text{RS}} - {\text{N}}_{\text{NRS}} )}} + \delta^{15} {\text{N}}_{\text{NRS}}$$

After we had δ^15^N_depo_, a similar mixing model equation was used for calculating the proportion of rhizo-deposition N in neighboring grass N (${\text{P}}_{{{\text{non - leg(}} \Leftarrow {\text{depo)}}}}$):

4 $${\text{P}}_{{{\text{non-leg}}( \Leftarrow {\text{depo}})}} = \frac{{\left( {\delta^{15} {\text{N}}_{\text{non-leg(mix)}} - \delta^{15} {\text{N}}_{\text{non-leg(ref)}} } \right)}}{{\left( {\delta^{15} {\text{N}}_{\text{depo}} - \delta^{15} {\text{N}}_{\text{non-leg(ref)}} } \right)}}$$

where ${\text{P}}_{{{\text{non-leg(}} \Leftarrow {\text{depo)}}}}$, the proportion of non-legume N derived from the transfer of root rhizo-deposition N, δ^15^N_non-leg (ref)_ is the natural ^15^N abundance of the shoot of the grass without any influence of legumes, and δ^15^N_non-leg (mix)_ is the natural ^15^N abundance of the shoot of the grass when grown with legumes.

### Data analysis

To eliminate the potential influence of variations in shrub size, we defined our six target shrub clusters as blocks. Data were tested for normality using the Shapiro–Wilk test and for equality of variances using Levene’s test. We used the *lme4* package (Bates et al. 2012) to perform a linear mixed model analysis for the response variable (plant N concentrations, plant δ^15^N, respectively). For each response variable model, species and distance were fixed effects while block (target shrub) was a random effect. Species-specific data were analyzed separately to assess the distance effect on each response variable. Visual inspection of residual plots did not reveal any obvious deviations from homoscedasticity or normality. *P* values were obtained through likelihood ratio tests of the full model with the effect in question against the model without this effect. The Tukey–Kramer test was used to address the presence of missing data (target grasses might not be present for one or two shrub clusters at some sampling distances, therefore for *L. chinensis* at distance 0 and 500 cm, we only have 5 and 4 replicates respectively; for *A. sibiricum* at distance 0 and 20 cm, we only have 5 and 4 replicates, respectively. *n* = 6 for others) for pairwise comparisons between different distances for each species. Differences were considered statistically significant when *P* < 0.05. Linear regressions were used to test the relationships for plant δ^15^N among each two of the three target species. Pairwise comparisons (paired *t* test) was used to test the shrub effect on plant biomass, individual height and numbers between plant community “Within” and “Open”. All these analysis were performed on R 2.15.3 software (R Development Core Team, http://www.r-project.org) (R Core Team 2013).

## Results

### Plant N concentration patterns with distance to C. microphylla

**Fig. 1:**
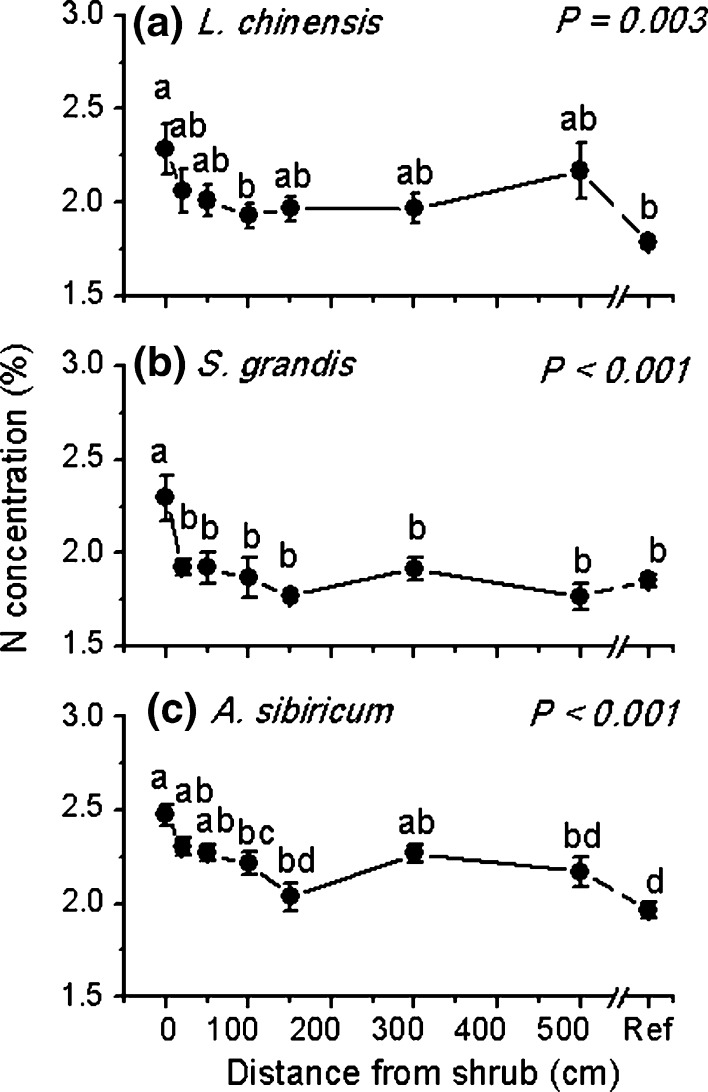
Nitrogen concentration (%) for three grass species, **a** *Leymus chinensis*, **b** *Stipa grandis*, and **c** *Achnatherum sibiricum*, in relation to distance from the leguminous shrub *Caragana microphylla.* *Ref* is for samples taken from an area where each species was assumed not to be influenced by the shrub. *P* values in *panels* indicate the distance effect. Values are mean ± SE. *Letters* indicate significant differences between different distances (0, 20, 50, 100, 150, 300, 500 cm and Ref) to the shrub (*P* < 0.05). Note that for *L. chinensis* at distances 0 and 500 cm, we only have 5 and 4 replicates, respectively; for *A. sibiricum* at distance 0 and 20 cm, we only have 5 and 4 replicates, respectively. *n* = 6 for others

Plant shoot N concentration was negatively correlated with the distance to the shrub (*L* = 77.71, *P* < 0.001) and strongly dependent on species identity (*L* = 71.93, *P* < 0.001; Table S1; Fig. 1). Specifically, the perennial rhizomatous grass *L. chinensis* around the shrub (distance up to 500 cm) had greater N concentration than those at the reference position (Fig. 1a). The N concentration of the perennial bunchgrass *S. grandis* in the immediate shrub area (i.e. distance = 0 cm) was significantly higher than that outside the shrub (i.e. distance > 0 cm) and the reference point (Fig. 1b). The N concentrations of *A. sibiricum* (the perennial bunchgrass with endophytic fungi) decreased with transect distance to the 150 cm position, beyond which N concentration was not significantly different from the reference (Fig. 1c).

### Plant δ^15^N patterns with distance to C. microphylla

**Fig. 2:**
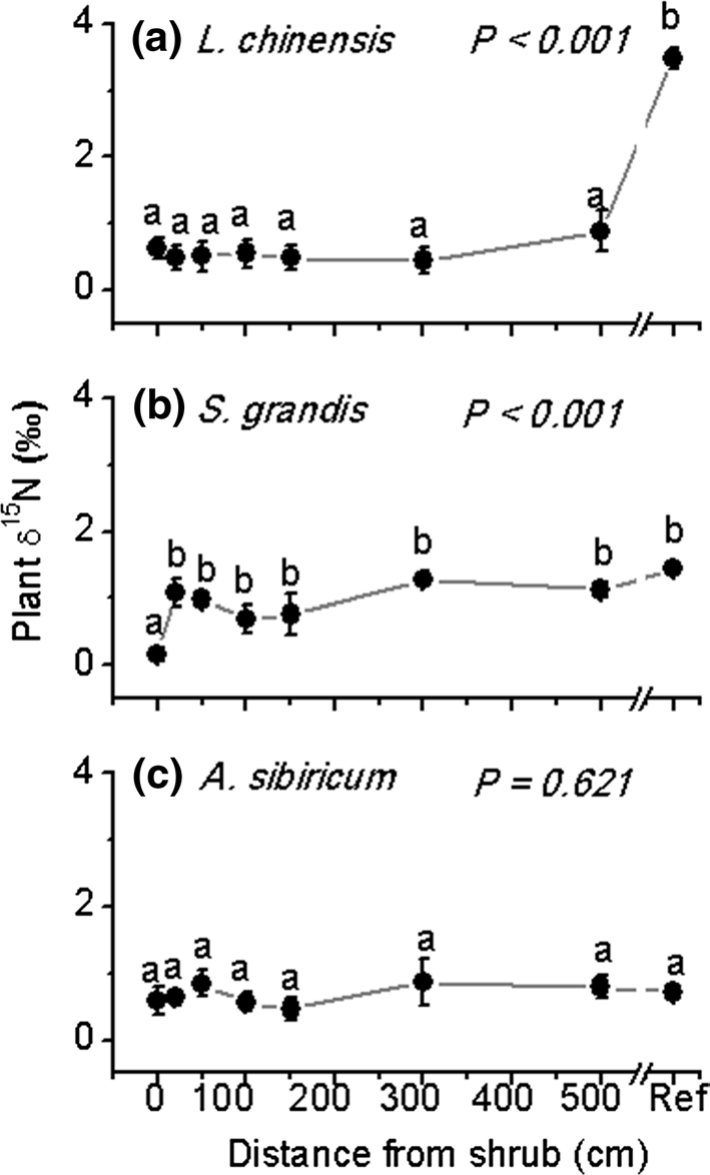
Plant δ^15^N for three grass species, **a** *Leymus chinensis*, **b** *Stipa grandis*, and **c** *Achnatherum sibiricum*, in relation to distance from the leguminous shrub *Caragana microphylla.* *Ref* is for samples taken from an area where each species was assumed not to be influenced by the shrub. *P* values in panels indicate the distance effect. Values are mean ± SE. *Letters* indicate significant differences between different distances (0, 20, 50, 100, 150, 300, 500 cm and Ref) to the shrub (*P* < 0.05). Note that for *L. chinensis* at distances 0 and 500 cm, we only have 5 and 4 replicates, respectively; for *A. sibiricum* at distance 0 and 20 cm, we only have 5 and 4 replicates, respectively. *n* = 6 for others

Shoot δ^15^N values mostly ranged between 0 and +1 ‰, and δ^15^N values increased with distance from the leguminous shrub (*L* = 53.53, *P* < 0.001), indicating plants benefitted more from N fixed by legumes. Spatial patterns of ^15^N with distance depended strongly on species identity (*L* = 116.52, *P* < 0.001; Table S1; Fig. 2). Specifically, plant δ^15^N for *L. chinensis* (*L* = 89.52, *P* < 0.001) were lower over the 500-cm transect away from the shrub compared with that from the reference point, which had a significantly more enriched ^15^N value (close to 4 ‰) (Fig. 2a). For *S. grandis*, δ ^15^N was significantly lower only at the 0 cm position, with other distances comparable to the reference point; Fig. 2b). *A. sibiricum* (*L* = 5.32, *P* = 0.621) showed no significant trend in δ ^15^N with distance (Table S2; Fig. 2c).

### Soil N and δ^15^N values with distance to C. microphylla

**Fig. 3:**
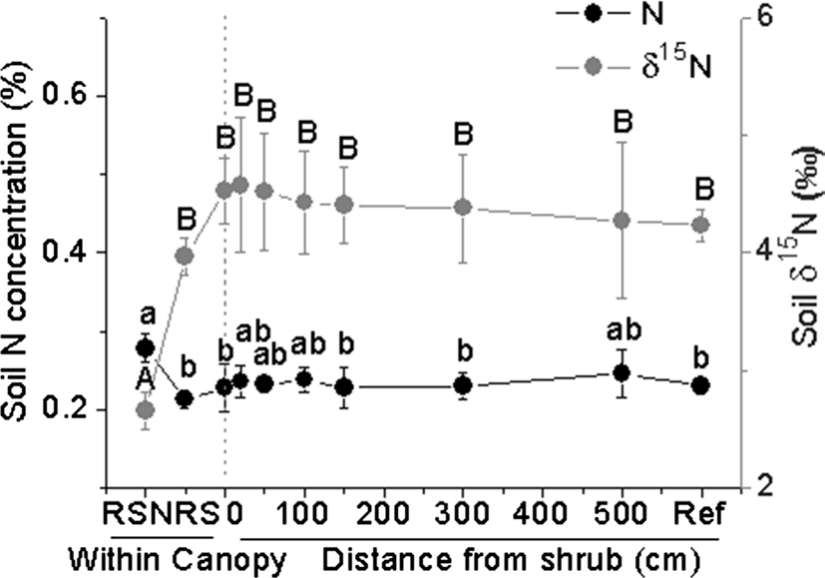
Soil N (%) and soil δ^15^N for rhizosphere soil (*RS*), non-rhizosphere soil (*NRS*), and eight distances from shrub *Caragana microphylla*.* Ref* is for soil samples taken from an area where assuming without any influence by the shrub. Soil was collected from grass roots with no species differentiation. Values are mean ± SE (*n* = 6). *Letters* indicate significant differences among different distances (RS, NRS, 0, 20, 50, 100, 150, 300, 500 cm and Ref) to the shrub (*P* < 0.05)

Neither soil N nor δ^15^N varied with distance to *C. microphylla* over a range from 0 to 500 cm, even extending to the reference site (15 m). However, soil N concentrations in the rhizosphere soil under *C. microphylla* plants was significantly greater than that of non-rhizosphere soil and also than that of the 0, 150, and 300 cm distances and the reference site. δ^15^N of the rhizosphere soil of *C. microphylla* was significantly lower than that of non-rhizosphere soils sampled other distances (Fig. 3).

### Proportion of N transferred to neighboring grasses from rhizo-deposition

**Fig. 4:**
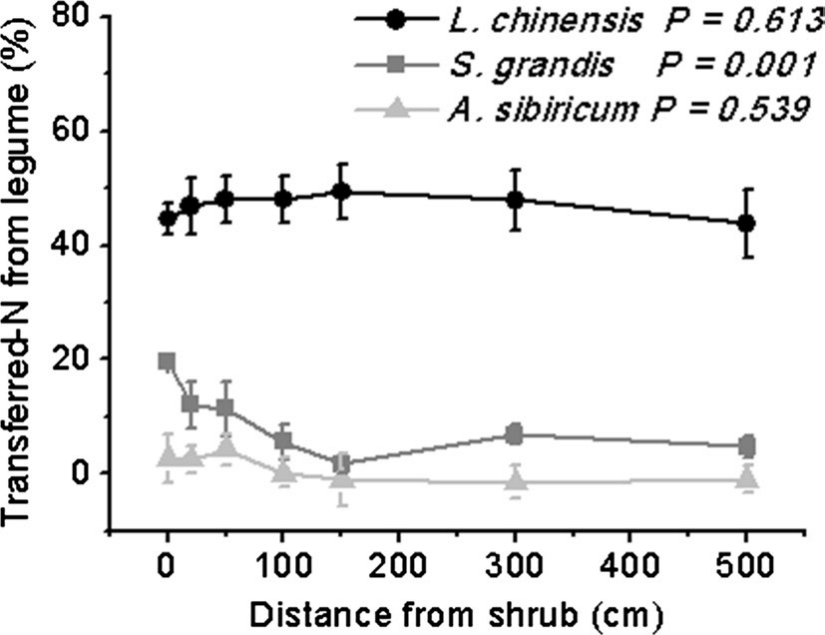
Estimated percentage of N transferred from rhizo-deposition to the three grass species, *Leymus chinensis*, *Stipa grandis*, and *Achnatherum sibiricum*, in relation to distance from the shrub *Caragana microphylla*. Values are mean ± SE, For *L. chinensis* at distances 0 and 500 cm, we only have 5 and 4 replicates, respectively; for *A. sibiricum* at distances 0 and 20 cm, we only have 5 and 4 replicates, respectively. *n* = 6 for others

The rhizo-deposition transferred N contributed ~47 % of the N in the perennial rhizomatous species *L. chinensis,* and this proportion remained constant across the 500-cm distances. Similarly, the rhizo-deposition transferred-N contributed only 1 % to the N of *A. sibiricum* (the perennial bunchgrass with endophytic fungi) and this proportion was also kept constant across the 500-cm distances (Fig. 4). The rhizo-deposition transferred N contributed to the N of the perennial bunchgrass *S. grandis* was up to 19 % only in the immediate shrub area (i.e. distance = 0 cm) and was not significant further away from the shrub (Fig. 4).

### Plant community structure and composition response to C. microphylla encroachment

**Fig. 5:**
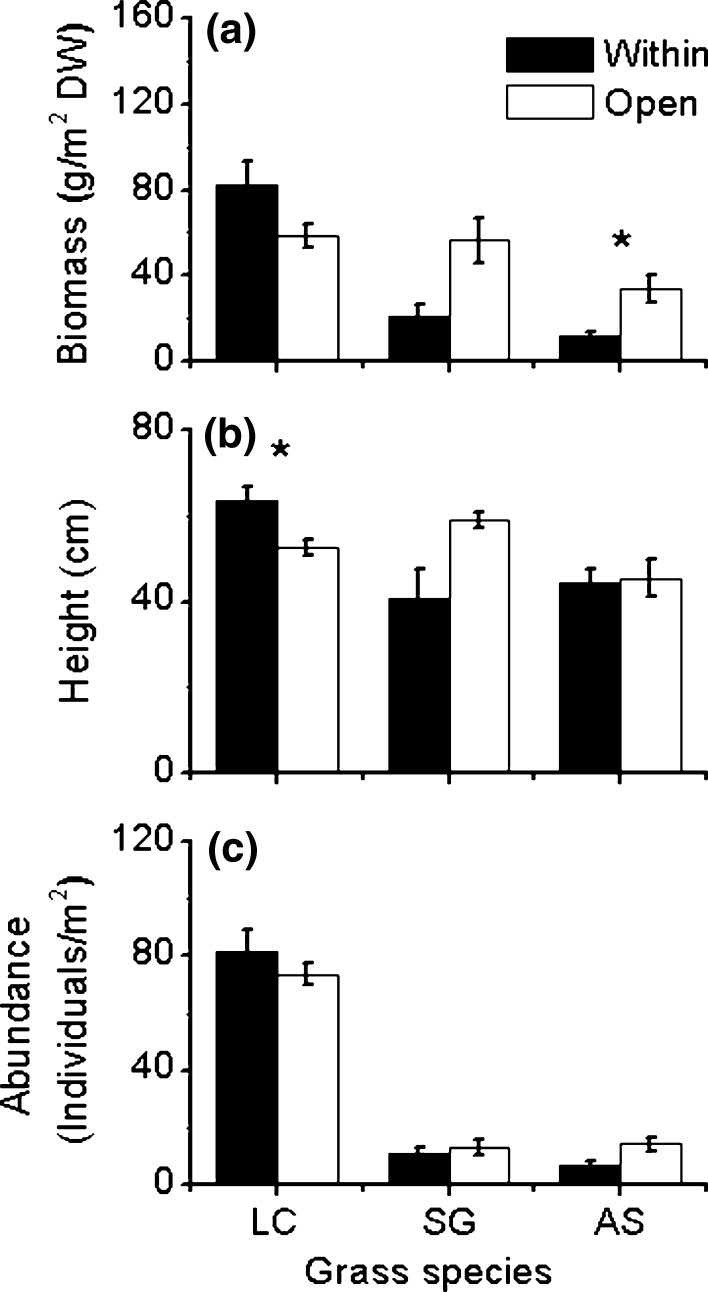
Size and density effects of *Caragana microphylla* on three grass species (*Leymus chinensis*,* LC*; *Stipa grandis*,* SC*; *Achnatherum sibiricum*,* AS*). Biomass (g/m^2^) dry weight (*DW*) (**a**), plant height (cm) (**b**) and abundances (individuals/m^2^) (**c**), of the dominant grasses at two positions relative to the shrub.* Within* is for samples taken within the canopy;* Open* is for samples taken outside the shrub (distance > 4 m). Values are mean ± SE (*n* = 6). Statistically significant effects of the positions are indicated by an asterisk (*p* < 0.05)

Shrub encroachment increased the height and shoot biomass of *L. chinensis* but decreased both for *S. grandis*, although only the effect on the height of *L. chinensis* was significant. However, shrub encroachment decreased shoot biomass for *A. sibiricum* but had no significant effect on their individual height (Fig. 5). For *L. chinensis* and *S. grandis*, shrub encroachment had no significant effect on the species abundance (e.g., number of individuals; Fig. 5). Overall, shrub encroachment significantly reduced the combined shoot biomass of all grass species but had no effect on that of forbs (Fig. S2), with a consequence of reducing species evenness (*P* = 0.02) within the shrub canopy (0.26 ± 0.06) compared to that of the open community (0.32 ± 0.06).

## Discussion

### Species-specific transfer of fixed N to neighbour grasses

We found that the presence of the legume shrub increased N concentration and decreased δ^15^N values of all grasses growing within the shrub canopy, implying a transfer of fixed N from the shrub to grasses (Temperton et al. 2007; Gubsch et al. 2011), and supporting our first hypothesis. Previous work has shown that the influence area of fixed N from a central legume is at least 3.5-fold greater than the physical area covered by the legume (Rascher et al. 2012), suggesting that legumes influence neighboring species beyond their canopy extent. Rasmussen et al. (2013) found that the horizontal fixed N transfer to grass exceeded 50 cm from clover by studying grass–clover mixtures. Here, we demonstrated that *C. microphylla* could influence the nutrient status of the perennial rhizomatous grass, *L. chinensis*, up to 500 cm distance from the shrub, i.e. 26 times greater than the shrub canopy area, indicating that the influence of a legume shrub can be much larger than we previously anticipated.

Direct root exudation and input from litter (both above- and below-ground) are two possible sources for N transfer from legumes to non-legume species (Haystead et al. 1988; Jalonen et al. 2009). Previous studies have shown that substantial N transfer between legume and non-legume plants still existed even when aboveground litter and residues are removed (Dulormne et al. 2003). Sierra and Nygren (2006) showed that N transfer in grasses correlated with legume root density, suggesting that fixed N was transferred mainly through root hair deposition or root exudation instead of via aboveground inputs (Jalonen et al. 2009). Nitrogenous low-molecular weight compounds such as amino acids are known to be released from living plant roots (Paynel et al. 2001). Neighboring plants can directly take up these compounds without prior mineralization by microbial organisms (Lipson and Näsholm 2001; Jones et al. 2004). For instance, white clover (legume) releases high proportions of fixed N through root exudation (part of which were in the form of ammonium) and can transfer 4 % of their fixed N to neighboring ryegrass within a two-month period (Lesuffleur et al. 2013). In our study, rhizosphere soil had higher N concentration and lower δ^15^N compared to non-rhizosphere soil (Fig. 3), indicating that root exudation or root hair input should be important sources for N-transfer from the leguminous shrub to neighboring grasses in this semi-arid grassland.

If the dominant source and the isotopic identity of the transferred N can be validated (Peoples et al. 2015), comparative measures of non-legume ^15^N with or without legume could suggest uptake the transferred-N and also provide a quantitative estimate of N transfer between plant species. Based on this, we assumed that all three grasses absorbed fixed N from the rhizo-deposition of the shrub and that the isotope signatures of the plant shoots offered a new method to quantify the proportion of transferred N from rhizo-deposition to the N of neighboring grasses.

While basing our measurements on comparisons of shoot δ^15^N and N concentration, we found that the traits associated with each grass helped explain the spatial distribution and extent of transferred N. The rhizomatous grass *L. chinensis* had δ^15^N derived from the legume shrub at least 500 cm from the plant, with no change in the proportion of transferred N (47 %) from rhizo-deposition of *C. microphylla* along the 500-cm distances. On the other hand, the bunchgrasses did not demonstrate such long-distance transport. Specifically, this proportion was only up to 19 % for *S. grandis* when growing within the shrub canopy and significantly reduced once away from the canopy, suggesting *S. grandis* only uptakes the fixed N within the shrub canopy (Fig. 4). Consistent with our findings, Pirhofer-Walzl et al. (2012) reported that grass with fibrous roots received greater amounts of N from legumes than forbs that have taproots based on a field ^15^N labeling experiment, suggesting species-specific differences in the amount of N transfer occurred for plants with different root traits (Chalk et al. 2014). Previous work has shown that N could be shared across all the ramets along a stolon of a rhizomatous grass, *Fragaria chiloensis*, though the large net transfers are only from nutrient-rich towards nutrient-poor sites (Stuefer et al. 1994; Alpert 1996). This nutrition sharing strategy seems likely to accelerate the growth and the spreading of clonal fragments away from the nutrition-rich site, and therefore lengthen the transfer distance. In this study, *L*. *chinensis*, by sharing the fixed N among ramets through connected stolons, had longer N transfer distances than *S. grandis*, which has individuals that bunch together within a very limited area. Together, our results suggest that root traits not only affect the fixed N transferring amount but also influence the overall distance of N transfer. Future studies should also measure ^15^N and N in root tissues, not only in shoots, to help understand mechanisms of N transfer and to ensure that whole-plant N sources are determined.

Of the grasses we studied, only *A. sibiricum* has the ability for 100 % infection with endophytic fungi (EF) (Wei et al. 2007). Our results showed that *A. sibiricum* did not uptake the fixed N from rhizo-deposition of the shrub directly. Inoculation with EF is associated with significant increases in total shoot N (Li et al. 2012), although the exact mechanism remains unclear (Newsham 2011). However, hyphae and microsclerotia (the structures formed by EF which is thought to be resting structures or propagules) in root cells lack a host-derived perifungal membrane for exchanging nutrients. Therefore, direct transfer of N via the hyphae of EF to their hosts cannot be a major mechanism for the increases of plant N (Peterson et al. 2008). The positive nutritional effects mediated by EF are only significant when roots are supplied with organic N, indicating the most probable mechanism for EF to enhance plant N status is by mineralizing organic compounds such as proteins, peptides and amino acids in the rhizosphere (Newsham 2011). In our study, higher N concentration of *A. sibiricum*, compared to the other two species (Fig. 1), might because the association with EF increases the ability to take up organic N, which is not available for the other two grasses. However, the exact N uptake source for *A. sibiricum* remains unclear and needs further investigation.

### Shrub-grass interactions were species-specific

Understanding the links between abiotic conditions and plant interactions is crucial for improving our understanding of plant community dynamics and has great implications for ecosystem management activities (Brooker 2006). In the present study, we found that shrub encroachment significantly increased N % of all the target grasses via transferring fixed N (Figs. 1, 2), but its ultimate effects (positive or negative) on the biomass of grasses were species-specific, rather than dependent only on N status (Fig. 3). Specifically, the shoot biomass for *L. chinensis* was greater when next to the shrub than that in an open area, whereas *S. grandis* showed an opposite trend, although only marginally significant (*P* < 0.1; Fig. 5). With no significant change in individual numbers for these two grasses, the dominant reason for the biomass changes would be the changes of plant height: *L. chinensis* became significantly taller when next to the shrub than when in an open area, thus alleviating light limitation under shrub canopy rather than being suppressed, which was the case for *S. grandis*. Plant biomass of *A. sibiricum* was also reduced by shrub encroachment (although only marginally significantly) with no change in plant average height but a reduction of plant individual numbers. The reason for growth suppression of *A. sibiricum* by the shrub is unclear, but might be because of less successful germination or seedling establishment under the shrub canopy. Overall, the magnitude of the net effects of shrub encroachment was highly species-specific. Thus, encroached leguminous shrub significantly affected community structure (Fig. 5; Fig. S2) and decreased plant evenness (supporting hypothesis 2). Our results suggest that the transfer of fixed N to neighboring grasses and the ultimate outcome of shrub–grass interactions were both highly species-specific, leading to a dramatic effect on plant community structure and composition under field conditions.

## Conclusions

Three co-occurring perennial grasses varied in tissue N and ^15^N, and also in the amount of fixed N transferred from the leguminous shrub and the distance that fixed N signal was transported. Perennial rhizomatous species can take up fixed N from the soil under the legume’s canopy and transfer it to other conspecific individuals up to 5 m away via rhizomes. Perennial bunchgrasses can only take up and transfer legume fixed N a limited distance. Similar spatial and temporal differences in inter- and intra-species N transfer have been observed in a range of different field sites (Rasmussen et al. 2013). Our work suggests that investigating the species-specific difference in fixed N uptake and transfer should focus on the belowground perspective, i.e. the perspective of belowground foraging for resources, will improve our understanding of observed changes in community composition and forage quality under scenarios of increasing shrub encroachment in both arid and semiarid grassland (Casper and Jackson 1997). However, the effect of leguminous shrubs on their neighboring grasses went beyond N subsides, and whether they can promote or suppress the neighboring plant growth were species-specific. This pattern suggests that the ultimate plant interaction should also depend on other factors, such as water and light availability, and microclimate. Comprehensive consideration of both the biotic and abiotic interactions is necessary for better predicting the responses of plant community structure and composition to shrub encroachment.

## Electronic supplementary material

Below is the link to the electronic supplementary material.

Supplementary material 1 (DOCX 558 kb)
