# Supplementary material for: Impacts of leguminous shrub encroachment on neighboring grasses include transfer of fixed nitrogen
Source: Oecologia. 2016 Jan 8;180:1213–22. doi: 10.1007/s00442-015-3538-5 (PMC4819502; doi:10.1007/s00442-015-3538-5)

**Supplemental Information**

**Table S1.** Summary of mixed-effects model analyses of plant N concentration and δ^15^N for grass species from 0.2 m × 0.2 m plots of seven (not including the distance for the reference plant) different distances to the shrub *Caragana microphylla*.

|  | | N concentration | | δ^15^N | |
| --- | --- | --- | --- | --- | --- |
|  | | *L* | *P* | *L* | *P* |
| Fixed effects | Distance (D) | **77.71** | **< 0.001** | **53.53** | **< 0.001** |
|  | Species (S) | **71.93** | **< 0.001** | 6.35 | 0.042 |
|  | D × S | 18.33 | 0.192 | **116.52** | **0.001** |

Significant effects for distance, species and their interaction were tested in separate model series after fitting the main effects. Models were fitted and compared by stepwise dropping fixed effects. Likelihood-ratio tests were applied to improve our model (*L* ratio) and test the statistical significance of the explanatory terms (*P* values). Significant effects are marked in bold.

**Table S2.** Summary of mixed-effects model analyses of distance effects on plant N concentration and δ^15^N ratio of each grass species from 0.2 m × 0.2 m plots along seven distances (not including the distance for the reference plant) to the shrub *Caragana microphylla*.

|  | | | N concentration | | δ^15^N | |
| --- | --- | --- | --- | --- | --- | --- |
|  | d.f. | | *L* | *P* | *L* | *P* |
| *L. chinensis* | | 6 | 21.64 | **0.003** | 89.52 | **< 0.001** |
| *S. grandis* | | 6 | 34.27 | **< 0.001** | 28.74 | **< 0.001** |
| *A. sibiricum* | | 6 | 44.13 | **< 0.001** | 5.32 | 0.621 |

Given are the degrees of freedom (d.f.), likelihood-ratio tests were applied to improve our model (*L* ratio) and test the statistical significance of the explanatory terms (*P* values). Significant effects are marked in bold.

**Fig. S1** Sampling schemes: four transects radiating outwards from the canopy centre of each target shrub in each cardinal direction. Seven 20 × 20 cm^2^ sampling quadrats centred at distances of 0, 20, 50, 100, 150, 300, and 500 cm (starting form the edge of the shrub canopy) were placed along each transect. For each same distance point of each target shrub, quadrats from four directions (4 in total) were pooled together representing one sample.

**Fig. S2** Biomass (g/m^2^ dry weight (DW)) of the grasses and forbs at two different positions from the shrub *Caragana microphylla.* Within (black) represents the position for within the shrub and open (grey) represents open area outside the shrub (distance > 4 m). Values are means ± 1 SE (n = 6). Statistically significant effects of the positions are shown with treatment letters (* if P < 0.05, ns if not significant).

**Fig. S1**


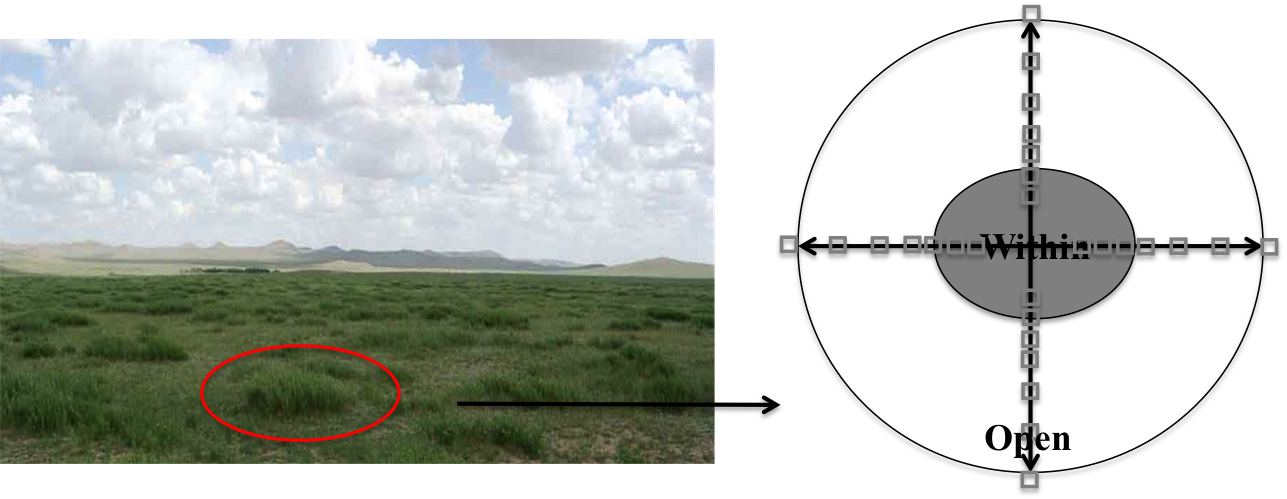


**Fig. S2**


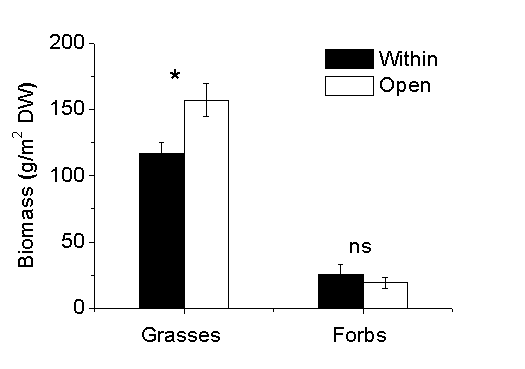

Supplement: Supplementary file 1 — Supplementary material 1 (DOCX 558 kb) [file 442_2015_3538_MOESM1_ESM.docx]
